# Supplementary material for: Food for thought: The impact of short term fasting on cognitive ability
Source: PLoS One. 2024 Nov 25;19(11):e0312811. doi: 10.1371/journal.pone.0312811 (PMC11588246; doi:10.1371/journal.pone.0312811)
Supplement: S1 Appendix — (PDF) [file pone.0312811.s001.pdf]

Appendix

Compliance With Treatment

Respondents were randomized into control and treatment (*3 and 12-hour fasting*) groups. Those in the *short* fasting treatment were instructed to fast for about 3 hours before coming to their experimental session, while those in the *long* fasting treatment were instructed to fast for at least 9 hours and not to eat after dinner the night before the experiment. However, since many of the initial sessions of the experiment took place at 8, 9 and 10am, we found that many participants in both treatments had not eaten since the previous night. Fig **??** demonstrates the self-reported fasting times for our two experimental groups comprised of 91 participants in the *short* fasting and 79 in the *long* fasting treatments.

S1 Fig. *3-hr* (left) and *12-hr* (right) Treatment Self-Reported Fasting. Note: Vertical Lines show Fasting Requirement.

While the majority of our experimental participants in the *long* fasting treatment group did comply with the suggested fast length of 9 hours, the majority of participants in the *short* fasting treatment also fasted for 9+ hours. As a result, we also conduct analysis with self reported fasting time as the independent variable of interest rather than treatment status.

Implicit Association Testing

We hypothesized that individuals' implicit associations between unhealthy and tasty food might act as a mediator of the relationship between short term fasting and cognitive ability. However, since we do not find any significant main effect to mediate, implicit association is instead retained as a control variable in our analysis. During the Implicit Association task, respondents were asked to categorize foods into combinations of categories and attributes:

S1 Table. Categories, Attributes, and Items used for IAT

Category			
Tasty	Untasty	Healthy	Unhealthy
Appealing	Bland	Apple	Cheeseburger
Appetizing	Disliked	Broccoli	Chips
Delicious	Flavorless	Chicken	Doughnut
Flavorful	Less Tasty	Salad	French Fries
Mouthwatering	Unappealing	Salmon	Pizza
Tasty	Unappetizing		
Yummy	Unpalatable		

Robustness

Most experimental sessions were conducted in the morning. As a result, assignment into the two fasting treatments did not create a significant difference in the average fasting hours between them. Many respondents in the 3-hour fast did not wake up and eat at least 3-hours before the session, resulting in fast times closer to the 12-hour overnight fasting group. In our main analysis the treated groups are combined into one category for analysis. As a robustness check, we dis-aggregate the treatment groups in columns

(1) and (2) of Table **??**. We provide two additional specifications for robustness (3) and (4) using total fasting hours rather than the treatment assignment.

S2 Table. Regressions on Raven's Test Score

	(1) Raven's Score	(2) Raven's Score	(3) Raven's Score	(4) Raven's Score
3-hr Fast Treatment	0.052*	0.048*		
	(0.030)	(0.026)		
12-hr Fast Treatment	0.011	0.020		
	(0.032)	(0.027)		
Fasting Hours			-0.001	-0.001
			(0.002)	(0.002)
Male		0.065***		0.069***
		(0.022)		(0.022)
Age		-0.008***		-0.008***
		(0.002)		(0.002)
Asian		0.003		0.005
		(0.031)		(0.030)
Hispanic		-0.113***		-0.110***
		(0.026)		(0.026)
Black + Other		-0.128***		-0.127***
		(0.046)		(0.048)
BMI		0.001		0.001
		(0.002)		(0.002)
Income		-0.001		-0.001
		(0.001)		(0.001)
Exercise		0.005		0.005
		(0.006)		(0.006)
Sleep		0.007		0.005
		(0.007)		(0.007)
IAT D-Score		-0.029		-0.023
		(0.018)		(0.018)
Constant	-0.336***	-0.200**	-0.308***	-0.151
	(0.024)	(0.101)	(0.021)	(0.097)
N	245	243	244	243

Robust Standard errors in parentheses
* $p < .1$, ** $p < .05$, *** $p < .01$
